# Prognostic utility of estimated albumin excretion rate in chronic kidney disease: results from the Study of Heart and Renal Protection

**DOI:** 10.1093/ndt/gfw396

**Published:** 2017-01-14

**Authors:** Marion M Mafham, Natalie Staplin, Jonathan Emberson, Richard Haynes, William Herrington, Christina Reith, Christoph Wanner, Robert Walker, Alan Cass, Adeera Levin, Bengt Fellström, Lixin Jiang, Hallvard Holdaas, Bertram Kasiske, David C Wheeler, Martin J Landray, Colin Baigent, the SHARP Collaborative Group

**Affiliations:** 1 Clinical Trial Service Unit and Epidemiological Studies Unit, Nuffield Department of Population Health, Oxford, UK; 2 Department of Medicine 1, Division of Nephrology, University of Wuerzburg, Wuerzburg, Germany; 3 Dunedin School of Medicine, University of Otago, Otago, New Zealand; 4 Menzies School of Health Research, Charles Darwin University, Darwin, Australia; 5 University of British Columbia, Vancouver, British Columbia, Canada; 6 University Hospital, Uppsala, Sweden; 7 National Clinical Research Centre of Cardiovascular Diseases, State Key Laboratory of Cardiovascular Disease, Fuwai Hospital, Chinese Academy of Medical Sciences and Peking Union Medical College, Beijing, People’s Republic of China; 8 Renal Section, Department of Transplant Medicine, Oslo University Hospital, Rikshospitalet, Oslo, Norway; 9 Hennepin County Medical Centre and the University of Minnesota, Minneapolis, MN, USA; 10 University College London, London, UK

**Keywords:** albuminuria, cardiovascular, chronic kidney disease, ESRD, mortality

## Abstract

**Background:**

Estimated albumin excretion rate (eAER) provides a better estimate of 24-h albuminuria than albumin:creatinine ratio (ACR). However, whether eAER is superior to ACR in predicting end-stage renal disease (ESRD), vascular events (VEs) or death is uncertain.

**Methods:**

The prognostic utility of ACR and eAER (estimated from ACR, sex, age and race) to predict mortality, ESRD and VEs was compared using Cox proportional hazards regression among 5552 participants with chronic kidney disease in the Study of Heart and Renal Protection, who were not on dialysis at baseline.

**Results:**

During a median follow-up of 4.8 years, 1959 participants developed ESRD, 1204 had a VE and 1130 died (641 from a non-vascular, 369 from a vascular and 120 from an unknown cause). After adjustment for age, sex and eGFR, both ACR and eAER were strongly and similarly associated with ESRD risk. The average relative risk (RR) per 10-fold higher level was 2.70 (95% confidence interval 2.45–2.98) for ACR and 2.67 (2.43–2.94) for eAER. Neither ACR nor eAER provided any additional prognostic information for ESRD risk over and above the other. For VEs, there were modest positive associations between both ACR and eAER and risk [adjusted RR per 10-fold higher level 1.37 (1.22–1.53) for ACR and 1.36 (1.22–1.52) for eAER]. Again, neither measure added prognostic information over and above the other. Similar results were observed when ACR and eAER were related to vascular mortality [RR per 10-fold higher level: 1.64 (1.33–2.03) and 1.62 (1.32–2.00), respectively] or to non-vascular mortality [1.53 (1.31–1.79) and 1.50 (1.29–1.76), respectively].

**Conclusions:**

In this study, eAER did not improve risk prediction of ESRD, VEs or mortality.

## INTRODUCTION

People with chronic kidney disease (CKD) face two major hazards: premature morbidity and mortality (in particular from cardiovascular disease) and progression to end-stage renal disease (ESRD) [1]. Measures of kidney function [e.g. estimated glomerular filtration rate (eGFR)] and markers of kidney damage (e.g. albuminuria) are associated with the risk of ESRD, vascular disease and death, both in the general population [2] and among those with CKD [3], although both are more strongly associated with ESRD than with vascular events (VEs) and death [4, 5].

Albuminuria is traditionally measured by timed urine collection but current guidelines recommend the measurement of albumin: creatinine ratio (ACR) in a spot urine sample, because ACR provides a reasonably accurate indication of albuminuria and is more convenient for patients [6, 7]. The ACR uses urine creatinine concentration as the denominator to account for urine concentration, but, since urine creatinine concentration is also affected by muscle mass, ACR may not reflect albuminuria accurately in some individuals [8].

Recently, equations have been developed to estimate the urine creatinine excretion rate (eCER) from age, sex and race (and weight in some cases) so that the ACR can be adjusted for factors related to muscle mass by calculating the estimated albumin excretion rate (eAER). Studies have shown that eAER estimates 24-h albumin excretion more accurately than ACR alone [9–11]. However, whether these measures provide additional prognostic information (i.e. improved ability to predict the risk of a future outcome) over ACR for ESRD, VEs or mortality is not known. Using data from 5552 participants with CKD in the Study of Heart of Renal Protection (SHARP) who were not on dialysis at study entry [12], we assessed whether eAER provides superior prognostic information to ACR alone.

## MATERIALS AND METHODS

The SHARP trial investigated the efficacy of lowering low-density lipoprotein cholesterol (LDL-C) with simvastatin/ezetimibe in 9270 participants with CKD (of whom 6245 were not on dialysis at randomization) [12]. The trial methods have been published in detail elsewhere and are summarized below [12, 13]. Ethics committee approval was obtained from all sites prior to enrolment and the study was conducted in accordance with the Declaration of Helsinki. SHARP was registered at ClinicalTrials.gov (NCT00125593) on 29 July 2005.

### Study participants

**FIGURE 1:**
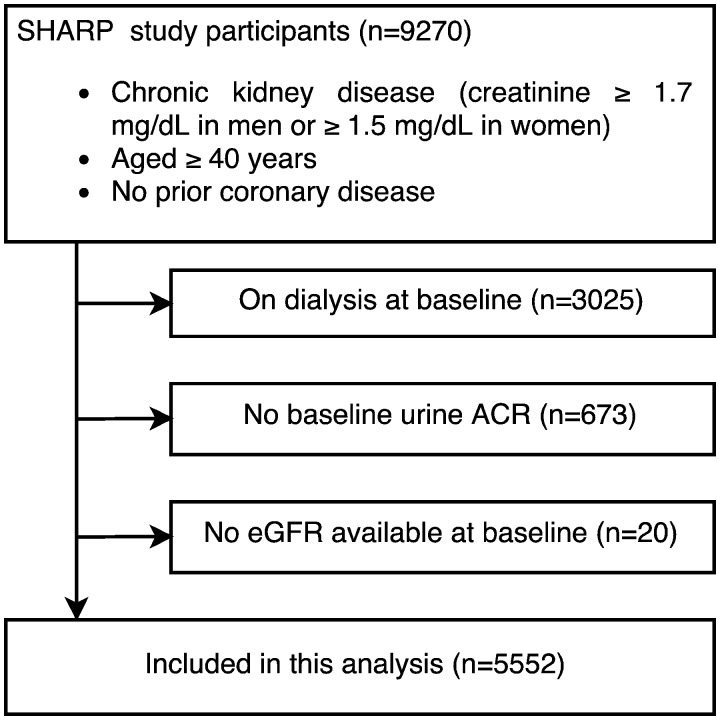
Eligibly criteria and participant selection. ACR, albumin creatinine ratio; eGFR, estimated glomerular filtration rate; SHARP, Study of Heart and Renal Protection.

Individuals aged 40 years or over were eligible to participate in SHARP if they had CKD with more than one previous measurement of serum or plasma creatinine of at least 1.7 mg/dL in men or 1.5 mg/dL in women. Participants with prior myocardial infarction or coronary revascularization were excluded. All participants provided informed consent prior to enrolment in the trial. Among the 6245 individuals not on dialysis at baseline, no baseline urine ACR was available for 673 participants and a further 20 had missing baseline central eGFR, leaving 5552 participants for analysis in this report (Figure 1).

### Baseline assessment

Self-reported history of prior vascular disease, diabetes, smoking status, race, co-medication and cause of kidney disease were recorded by trained study staff. The recorded cause of kidney disease was based on the clinical diagnosis of the managing physician and subsequently categorized into four groups: glomerulonephritis, diabetic nephropathy, cystic kidney disease and other causes (including unknown) [14]. Blood pressure, height and weight were measured by the study staff.

### Laboratory methods

Samples of non-fasting blood and urine (whenever in the day the visit occurred) were collected from all participants. Blood samples were cooled, centrifuged and separated, before being stored locally at −40 °C. Samples were then shipped on dry ice to the central laboratory in Oxford where assays of plasma creatinine and urine ACR were conducted. Creatinine and albumin were measured using a Synchron LX20 or DXC800 analyser (Beckman Coulter, Brea, CA, USA). Creatinine was assayed using a kinetic alkaline picrate method, calibrated using material traceable to National Institute of Standards and Technology Standard Reference Material 914a, with a mean expanded uncertainty of 13.4% (7.3% excluding biological variation). eGFR was calculated using the Chronic Kidney Disease Epidemiology Collaboration (CKD-EPI) formula [15]. eAER was calculated by:

eAER (mg/day) = ACR (mg/g)×eCER (g/day).

The eCER was calculated using the formula developed from the Modification of Diet in Renal Disease (MDRD) study as this did not require knowledge of the individual’s weight and so is more likely to be used in clinical practice (eAER_Ellam_) [9]. Sensitivity analyses were conducted using other eCER formulae (eAER_Ix_ and eAER_Walser_) [10, 11], restricted to the 5522 participants with information on weight at baseline. The eCER formulae are provided in the Supplementary data.

### Follow-up

Participants were to be seen at 2 and 6 months after randomization, and then every 6 months until final follow-up. Information on all serious adverse events was sought at each visit and further documentation collected on events of interest (including initiation of renal replacement therapy and possible VEs) by study staff. This information was sent to the international coordinating centre, in Oxford, for central adjudication. A non-fasting blood and urine sample for central laboratory analysis was requested from all participants known to be alive at 2.5 years after randomization.

The main outcomes of interest for these analyses were ESRD, VEs, vascular mortality and non-vascular mortality. The outcome of VEs used in this analysis includes a broader range of events than the pre-specified outcome used for the main trial results [12]. For this analysis, VEs include any cardiovascular death, non-fatal myocardial infarction, hospitalization with angina, any stroke, arterial revascularization, heart failure, arrhythmia or valvular heart disease (see Supplementary data). ESRD was defined as initiation of maintenance dialysis or renal transplantation.

### Statistical analysis

Cox proportional hazards regression was used to compare the relevance of ACR and eAER to the risk of ESRD, VEs and mortality over the period studied. The proportional hazard assumption was tested through examination of the time-dependency of the Schoenfeld partial residuals. The analyses were adjusted for baseline age, sex and eGFR. Analyses of VEs and mortality were also adjusted for other established cardiovascular risk factors [ethnicity, country, systolic and diastolic blood pressure, LDL-C, high-density lipoprotein cholesterol (HDL-C), smoking status, prior diabetes and prior vascular disease]. There were complete data on all these variables with the exception of systolic and diastolic blood pressure, LDL-C and HDL-C, for which 17 (0.3%) participants were missing at least one measurement. Median values were imputed for these participants.

The analyses relate risk to the estimated ‘usual’ ACR or eAER to correct for the regression dilution bias that would be introduced if only the baseline values were used [16]. In the figures, relative risks (RRs: approximated by the hazard ratio estimates from the Cox models) for each fifth of ‘usual’ albuminuria measure, including that for the reference group, are accompanied by a confidence interval (CI) derived from the variance of the log risk in that group. These group-specific CIs can be thought of as reflecting the amount of data only in that one group, thereby allowing appropriate statistical comparisons to be made between any two groups [17]. For ESRD, the top fifth is further divided into two equally sized groups, giving six comparison groups. The association between each measure of albuminuria with the outcomes of interest is summarized as the RR per 10-fold increase in albuminuria marker, as this allows comparison with other studies [18] and roughly equates to an increase in albuminuria stage [6]. The magnitude of improvement in risk prediction (over and above other baseline characteristics in the model) was estimated by the difference in twice the log-likelihood statistic between the two “nested” models [which, under the null hypothesis of no improvement, gives a chi-squared (χ^2^) statistic with 1 degree of freedom]. This provides not only a test for improvement in fit, but also a quantitative measure of the extent to which the added term improves risk prediction [19] and is the uniformly most powerful test of the incremental value of a biomarker [20]. A statistically significant improvement is indicated by a change in χ^2^ of at least 3.84 (for 1 degree of freedom). As a sensitivity analysis, analyses for ESRD were repeated using Fine and Gray regression, which yields sub-distribution hazard ratios [21], to account for the competing risk of death before ESRD.

## RESULTS

Average baseline characteristics in five groups defined by the quintiles of baseline urine ACR are shown in Table 1. Compared with participants with lower urine ACR, individuals with higher urine ACR were younger, had lower eGFR and higher blood pressure at baseline and were more likely to have a diabetic nephropathy or glomerulonephritis recorded as the cause of their renal disease (Table 1). The correlation between ACR or eAER measured at baseline and a repeat measurement (collected around 2.5 years later) was 0.75.

**Table 1:** Baseline demographic characteristics and physical/laboratory measurements among 5552 patients not on dialysis at randomization, by ACR group defined by the quintiles of the distribution

	ACR (mg/g)					P-value for differences between groups
	<30 *n* = 1109	≥30 to <118 *n* = 1109	≥118 to <347 *n* = 1120	≥347 to <1012 *n* = 1102	≥1012 *n* = 1112	
Age at randomization (years)	66 (12)	64 (12)	62 (11)	61 (12)	60 (12)	<0.0001
Men	693 (62%)	688 (62%)	693 (62%)	712 (65%)	678 (61%)	0.48
Race						
White	909 (82%)	872 (79%)	788 (70%)	731 (66%)	625 (56%)	<0.0001
Black	18 (2%)	21 (2%)	16 (1%)	18 (2%)	17 (2%)	0.93
Asian	164 (15%)	195 (18%)	298 (27%)	319 (29%)	450 (40%)	<0.0001
Other/not specified	18 (2%)	21 (2%)	18 (2%)	34 (3%)	20 (2%)	0.07
Prior vascular disease	153 (14%)	179 (16%)	134 (12%)	141 (13%)	201 (18%)	0.0002
Diabetes	197 (18%)	176 (16%)	233 (21%)	249 (23%)	407 (37%)	<0.0001
Current smoker	89 (8%)	125 (11%)	139 (12%)	155 (14%)	181 (16%)	<0.0001
Systolic blood pressure (mmHg)	134 (20)	136 (19)	138 (20)	143 (20)	148 (22)	<0.0001
Diastolic blood pressure (mmHg)	77 (12)	79 (11)	79 (12)	82 (12)	83 (13)	<0.0001
Body mass index (kg/m^2^)	27.9 (5.3)	27.3 (5.1)	27.0 (5.2)	27.0 (5.3)	27.1 (5.7)	0.0003
Renal diagnosis						
Glomerulonephritis	92 (8%)	130 (12%)	211 (19%)	278 (25%)	262 (24%)	<0.0001
Diabetic nephropathy	91 (8%)	86 (8%)	135 (12%)	161 (15%)	319 (29%)	<0.0001
Cystic kidney disease	128 (12%)	203 (18%)	160 (14%)	87 (8%)	36 (3%)	<0.0001
Other diagnoses	744 (67%)	624 (56%)	584 (52%)	541 (49%)	466 (42%)	<0.0001
CKD-EPI eGFR (mL/min/1.73 m^2^)						
Mean (SD)	31.7 (13.4)	26.6 (12.3)	23.7 (11.9)	22.6 (11.6)	20.9 (12.0)	<0.0001
≥60	34 (3%)	16 (1%)	7 (1%)	5 (0%)	12 (1%)	<0.0001
≥30 to <60	540 (49%)	373 (34%)	300 (27%)	267 (24%)	221 (20%)	<0.0001
≥15 to <30	466 (42%)	534 (48%)	510 (46%)	502 (46%)	451 (41%)	0.0022
<15	69 (6%)	186 (17%)	303 (27%)	328 (30%)	428 (38%)	<0.0001
Urinary ACR (mg/g)	11 (6–19)	63 (44–88)	208 (155–265)	601 (457–764)	1866 (1325–3002)	<0.0001
eCER (mg/day)^a^	1329 (1007–1556)	1354 (1031–1578)	1370 (1038–1614)	1422 (1057–1630)	1416 (1057–1640)	<0.0001
eAER (mg/day)^a^	14 (8–24)	80 (54–114)	265 (201–352)	781 (589–1057)	2513 (1819–3983)	<0.0001

Data are *n* (%), mean (standard deviation) or median (interquartile range). ACR, albumin:creatinine ratio; eCER, estimated creatinine excretion rate; CKD-EPI, Chronic Kidney Disease Epidemiology Collaboration; eAER, estimated albumin excretion rate; eGFR, estimated glomerular filtration rate.

a Calculated using the Ellam equation for estimated creatinine excretion rate.

### Measures of albuminuria and the risk of ESRD

**FIGURE 2:**
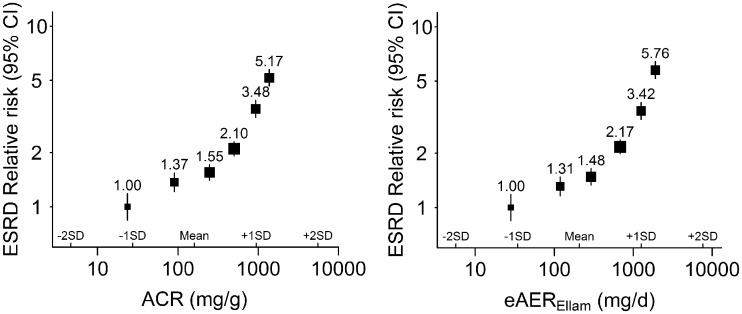
Relative risk of ESRD at different levels of ACR and eAER. ACR, albumin:creatinine ratio; eAER, estimated albumin excretion rate; ESRD, end-stage renal disease; SD, standard deviation. The relative risks (adjusted for age, sex and eGFR) and 95% confidence intervals are plotted against the mean usual value (i.e. the medium-term average value). Participants are split into five equally sized groups with the top fifth further divided equally in two.

After a median follow-up of 4.8 years among survivors, 1959 participants had reached ESRD. Both measures of albuminuria displayed strong associations with the risk of ESRD after adjustment for age, sex and eGFR (Figure 2). For both measures curvilinear relationships with ESRD were seen when plotted on a log-log scale, with stronger relationships observed among those with more albuminuria (Figure 2). The average RR throughout the range of values studied was nearly identical for the two measures: RR per 10-fold higher ACR 2.70 (95% CI 2.45–2.98); and per 10-fold higher eAER 2.67 (95% CI 2.43–2.94) (Table 2). Neither ACR nor eAER provided any additional prognostic information for ESRD risk over and above each other [improvement in risk prediction for eAER over and above ACR χ^1^_2_^ ^=^ ^0.3); for ACR over and above eAER ($\chi_{1}^{2}$^ ^=^ ^0.0); Supplementary Table S1].

**Table 2:** Age- and sex-adjusted relevance of each marker of albuminuria to ESRD, vascular events and mortality risk before and after additional adjustment for eGFR

Albuminuria marker	Relative risk per 10-fold increase in marker of albuminuria (95% CI)	
	Adjusted for age and sex	Adjusted for age, sex and eGFR
ESRD		
ACR	3.52 (3.22, 3.85)	2.70 (2.45, 2.98)
eAER_Ellam_	3.46 (3.17, 3.78)	2.67 (2.43, 2.94)
Vascular events		
ACR	1.53 (1.38, 1.70)	1.37 (1.22, 1.53)
eAER_Ellam_	1.53 (1.38, 1.69)	1.36 (1.22, 1.52)
Vascular mortality		
ACR	1.97 (1.63, 2.40)	1.64 (1.33, 2.03)
eAER_Ellam_	1.95 (1.61, 2.36)	1.63 (1.32, 2.00)
Non-vascular mortality		
ACR	1.88 (1.63, 2.18)	1.52 (1.30, 1.78)
eAER_Ellam_	1.86 (1.61, 2.15)	1.50 (1.29, 1.76)
All-cause mortality		
ACR	1.88 (1.69, 2.10)	1.56 (1.38, 1.75)
eAER_Ellam_	1.86 (1.67, 2.07)	1.54 (1.37, 1.73)

ACR, albumin:creatinine ratio; eAER, estimated albumin excretion rate; eGFR, estimated glomerular filtration rate; ESRD, end-stage renal disease; CI, confidence interval.

The predictive power of ACR and eAER for ESRD was also similar in various subgroups; in men and women; among White and Asian participants; among those with an eGFR ≥30 mL/min/1.73 m^2^ and eGFR <30 mL/min/1.73 m^2^ at baseline; and when the population was separated into groups by age, weight or blood pressure (Supplementary Table S2). The prognostic utility of eAER was tested separately in the four groups of cause of kidney disease (glomerulonephritis, diabetic nephropathy, cystic kidney disease and other causes; Supplementary Table S2). In each category of renal disease, a model including either ACR or eAER, along with age, sex and eGFR, was just as informative as one using both measures (χ^2^ with either ACR or eAER was 98–99% of the χ^2^ for both) although in those with cystic kidney disease neither measure of albuminuria was particularly predictive (χ^2 ^=^ ^4.6 for the model including ACR and eAER).

In a model without adjustment for age, sex or eGFR, eAER did not add any additional prognostic information for ESRD risk over and above ACR ($\chi_{1}^{2}$ = 3.8), but ACR did add prognostic information over and above eAER ($\chi_{1}^{2}\;$= 38.3) (Supplementary Table S1). The overall results were similar when eAER was calculated using formulae that include weight as well as age, sex and race (Supplementary Table S1) [10, 11]. Additionally, neither ACR nor eAER provided any additional prognostic information for ESRD risk over and above each other when accounting for the competing risk of death before ESRD (Supplementary Table S3).

### Measures of albuminuria and the risk of VEs

**FIGURE 3:**
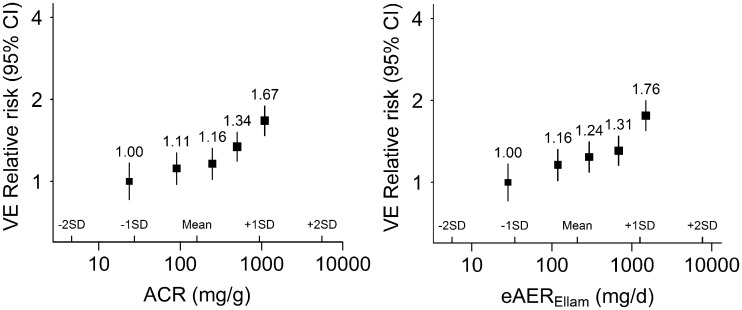
Relative risk of vascular events at different levels of ACR and eAER. ACR, albumin:creatinine ratio; eAER, estimated albumin excretion rate; VE, vascular events; SD, standard deviation. The relative risks (adjusted for established risk factors and eGFR) and 95% confidence intervals are plotted against the mean usual value (i.e. the medium-term average value). The established risk factors included are: age, sex, ethnicity, country, systolic blood pressure, diastolic blood pressure, low-density lipoprotein cholesterol, high-density lipoprotein cholesterol, smoking status, prior diabetes and prior vascular disease.

During follow-up 1204 participants suffered a fatal or non-fatal VE. Baseline urine ACR and eAER_Ellam_ had modest positive associations with the risk of VEs after adjustment for established cardiovascular risk factors and eGFR (Figure 3), with almost identical RRs: RR per 10-fold higher ACR 1.37 (95% CI 1.22–1.53); and per 10-fold higher eAER 1.36 (1.22–1.52) (Table 2). Again, neither ACR or eAER provided any additional prognostic information for VE risk over and above each other [improvement in risk prediction for eAER over and above ACR ($\chi_{1}^{2}$^ ^ = ^ ^0.8); for ACR over and above eAER ($\chi_{1}^{2}$^ ^= ^ ^0.5); Supplementary Table S4].

Findings were similar when eAER was calculated using the formulae that included weight (Supplementary Table S4) [10, 11].

### Measures of albuminuria and mortality

**FIGURE 4:**
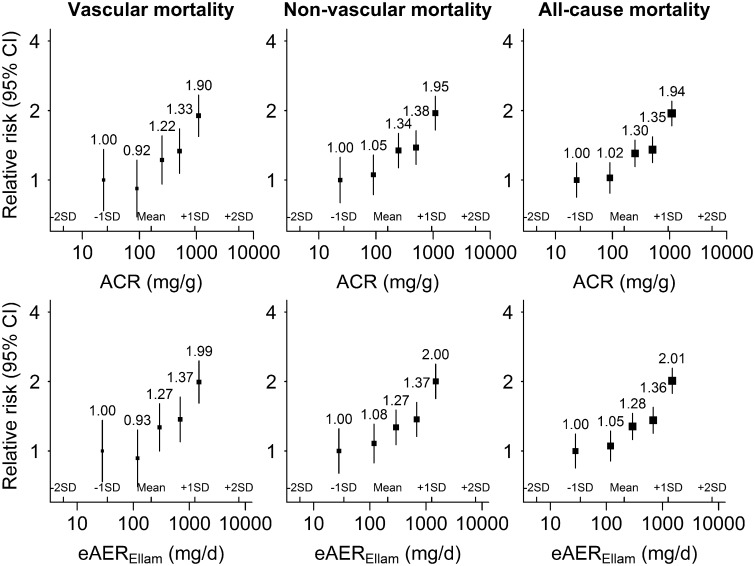
Relative risk of mortality at different levels of ACR and eAER_Ellam_, after adjustment for established risk factors and estimated glomerular filtration rate. ACR, albumin:creatinine ratio; eAER, estimated albumin excretion rate; SD, standard deviation. The relative risks and 95% confidence intervals are plotted against the mean usual value (i.e. the medium-term average value). The established risk factors included are: age, sex, ethnicity, country, systolic blood pressure, diastolic blood pressure, low-density lipoprotein cholesterol, high-density lipoprotein cholesterol, smoking status, prior diabetes and prior vascular disease.

In total, 1130 participants died during follow-up, 369 from a vascular cause and 641 from a non-vascular cause. The cause of death was not known in 120 participants. After adjustment for established risk factors and eGFR, both ACR and eAER_Ellam_ showed modest associations with both vascular and non-vascular mortality, and consequently all-cause mortality (Figure 4). After adjustment for known cardiovascular risk factors and eGFR, a 10-fold higher ACR or eAER_Ellam_ was associated with almost identical RRs for vascular mortality [1.64 (1.33–2.03) and 1.63 (1.32–2.00), respectively], for non-vascular mortality [1.52 (1.30–1.78) and 1.50 (1.29–1.76), respectively] and hence for all-cause mortality [1.56 (1.38–1.75) and 1.54 (1.37–1.73), respectively] (Table 2).

For vascular mortality, neither measure significantly improved model fit over and above the other ($\chi_{1}^{2}$^ ^ = ^ ^2.0 for eAER over and above ACR and $\chi_{1}^{2}$ = ^ ^2.3 for ACR over and above eAER; Supplementary Table S5). For non-vascular mortality, however, knowledge of both albuminuria measures resulted in small, but significant, improvements in risk prediction compared with knowledge of either one in isolation $\chi_{1}^{2}$^ ^= ^ ^8.2 for eAER over and above ACR and $\chi_{1}^{2}$^ ^= ^ ^9.1 for ACR over and above eAER; Supplementary Table S5). Again, findings were similar when alternative equations that included weight [10, 11] were used to estimate eAER (Supplementary Table S5).

## DISCUSSION

Albuminuria is recognized as a strong predictor of the risk of ESRD and of VEs [2, 4]. This report confirms these associations but shows that eAER (a more accurate estimate of 24-h urine albumin excretion than ACR) does not provide more prognostic information than ACR in terms of ESRD or vascular risk. The likely explanation for this finding lies in relationships between ACR and eAER and measured albuminuria.

The eAER_Ellam_ formula was derived from measurements of 24-h urinary excretion of albumin and creatinine in the MDRD study [9]. One of the key findings from the derivation of this formula was that almost all of the MDRD participants, with the exception of White women, excreted substantially more than 1 g of creatinine per day. Thus, estimates of 24-h albuminuria based on ACR, which assumes the amount of albumin per gram of creatinine to be equivalent to the 24-h albumin excretion, are likely to systematically underestimate measured albuminuria in most individuals. This is consistent with the authors' finding that ACR underestimated albuminuria in both validation cohorts, whereas eAER produced a relatively unbiased estimate (assessed by the median difference between ACR or eAER and measured 24-h albuminuria) [9]. However, in the validation cohorts, eAER was not more precise than ACR, meaning that, after accounting for the bias, for any individual in the study the difference between the ACR or eAER estimate and measured 24-h albuminuria was similar (assessed by the interquartile range of the differences between ACR or eAER and measured albuminuria). Because accuracy (assessed as the proportion of individuals in whom ACR or eAER was within 30% or 50% of the measured albuminuria) is heavily dependent on the bias, eAER is substantially more accurate in predicting 24-h albuminuria than ACR [9]. In predicting events in a prospective study, however, the bias is less important than discrimination (i.e. the measure’s ability to order individuals according to their level of risk).

Since eAER aims to account for differences in creatinine excretion between individuals it might be expected to perform better than ACR in predicting ESRD without adjustment for other factors, including age and sex. However, in this study, eAER did not provide any additional prognostic information over ACR in predicting ESRD risk in either the null or the adjusted model. Furthermore, knowledge of eAER and ACR was no more informative than ACR alone in predicting ESRD in subgroups expected to have lower creatinine excretion (e.g. women, those aged over 70 years, Asian participants, those weighing under 70 kg) or higher creatinine excretion (e.g. men, those aged under 50 years, White participants and those weighing over 85 kg). It is also interesting that the formulae that included weight in the calculation of CER (eAER_Ix_ or eAER_Walser_), and therefore eAER, also did not provide additional prognostic information over ACR. However, similar to the eAER_Ellam_ equation, in the Prevention of Renal and Vascular Endstage Disease (PREVEND) cohort neither the eAER_Ix_ or the eAER_Walser_ formulae were more precise than ACR in predicting measured albuminuria, although they both produced a less biased estimate of albuminuria than ACR [10].

This current study has several important strengths. First, the large number of ESRD events mean that we were able to not only estimate the risks associated with higher albuminuria very precisely, but also compare the predictive performance of measures of albuminuria separately in a number of important subgroups. Second, the measurement of urine and blood biochemistries was conducted in a central laboratory using standard methods. Third, the study population had a wide range of albuminuria including over 1000 individuals with an ACR of >1 g/g.

A number of limitations require consideration. First, albuminuria was measured using a sample collected at only a single time-point. Within individuals, day-to-day variability of albuminuria is substantial [22] and the use of albuminuria measured at just a single time point would underestimate the strength of the true association between usual albuminuria and risk [16]. We were, to some extent, able to overcome this problem by correcting for the regression dilution bias [16]. The regression dilution ratio (the correlation between baseline ACR or eAER and a repeat measurement collected around 2.5 years later) was 0.75 for both albuminuria measures and therefore correction for this within-individual variation resulted in an association between usual ACR or eAER and risk of ESRD, VEs and mortality (reported here) that was approximately one-third stronger than would have been observed without correction [16]. Second, measures of 24-h albuminuria were not available and so we were not able to assess the prognostic ability of eAER compared with a ‘gold standard’. However, the aim of these analyses was to assess whether eAER is superior in terms of predicting ESRD or VEs to ACR, the albuminuria measure used in clinical practice [6]. Third, the study included only individuals with moderate to severe CKD and therefore it is possible that eAER might be more useful that ACR in predicting ESRD or VEs in the general population.

As a major use of ACR in clinical practice is to predict risk of progression of CKD (and to a lesser extent, risk of vascular disease), an important question is whether eAER provides superior prognostic information to ACR alone. These analyses show that if ACR is already known (as it would be in clinical practice), eAER provides no extra predictive prognostic information.

## SUPPLEMENTARY DATA

Supplementary data are available online at http://ndt.oxfordjournals.org.

## Supplementary Material

Supplementary material

Supplementary Technical appendix
