# Supplementary material for: Prognostic utility of estimated albumin excretion rate in chronic kidney disease: results from the Study of Heart and Renal Protection
Source: Nephrol Dial Transplant. 2017 Jan 14;33(2):257–64. doi: 10.1093/ndt/gfw396 (PMC5837220; doi:10.1093/ndt/gfw396)
Supplement: Supplementary material [file gfw396_2016-08-24_sharp_acr_vs_eaer_supplementary_material.pdf]

Supplementary material

Formulae used to calculate estimated creatinine excretion rate (eCER)

Ellam equation for eCER

$eCER_{ellam}$ (md/day) =

male/black: $1413.9 + (23.2 \times age) - (0.3 \times age^2)$

female/black: $1148.6 + (15.6 \times age) - (0.3 \times age^2)$

male/nonblack: $1307.3 + (23.1 \times age) - (0.3 \times age^2)$

female/nonblack: $1051.3 + (5.3 \times age) - (0.1 \times age^2)$

Ix Equation for eCER

$eCER_{Ix}$ (mg/day) = 879.89 + (12.51 x weight [kg]) – (6.19 x age) + 34.51 (if black) – 379.42 (if female)

Walser equation for eCER

$eCER_{walser}$ (mg/day) =

Male: (28.2 – 0.172 x age) x weight (kg)

Female: (21.9 – 0.115 x age) x weight (kg)

Definition of vascular events

In this analysis a vascular event included any of the following:

- Cardiovascular death
- Coronary events: Non-fatal myocardial infarction, unstable angina
- Cerebrovascular disease events: Non-fatal stroke (including haemorrhagic stroke and subarachnoid haemorrhage), transient ischaemic attack, amaurosis fugax, retinal artery occlusion
- Peripheral vascular disease events: Aortic aneurysm (including rupture and dissection), limb ischaemia, limb artery embolism or thrombosis
- Arterial revascularization: coronary or non-coronary (excluding interventions on haemodialysis access)
- Heart failure
- Arrhythmias: atrial fibrillation, ventricular tachycardia, other tachycardia, bradycardia/heart block, other arrhythmia, cardiac arrest
- Valvular heart disease (including valve repair or replacement) and pericardial disease (pericarditis, effusion

Deaths were considered to be vascular if they were caused by any of the vascular events described above, the remaining deaths were considered to be non-vascular unless the cause of death was not known.

Table S1: Comparison of predictive strengths for ESRD relative risk of different indicators of albuminuria, using alternative equations to estimate creatinine excretion rate.

	Relative risk per 10 fold increase in indicator of albuminuria (95% CI)	Degrees of freedom (df)	Improvement in fit (χ^2)§
Null model		0	0
plus ACR	3.67 (3.36,4.00)	1	976.8
plus $eAER_{Ellam}$	3.44 (3.16,3.74)	1	942.3
plus $eAER_{Ix}$	3.45 (3.17,3.76)	1	931.6
plus $eAER_{Walser}$	3.45 (3.17,3.75)	1	944.3
plus ACR and $eAER_{Ellam}$		2	980.6
plus ACR and $eAER_{Ix}$		2	980.5
plus ACR and $eAER_{Walser}$		2	977.0
Age and sex		2	150.5
plus ACR	3.52 (3.22,3.85)	3	1031.4
plus $eAER_{Ellam}$	3.46 (3.17,3.78)	3	1029.6
plus $eAER_{Ix}$	3.48 (3.18,3.80)	3	1023.7
plus $eAER_{Walser}$	3.43 (3.14,3.74)	3	1015.7
plus ACR and $eAER_{Ellam}$		4	1033.1
plus ACR and $eAER_{Ix}$		4	1031.5
plus ACR and $eAER_{Walser}$		4	1031.5
Age, sex and eGFR		3	2788.3
plus ACR	2.70 (2.45,2.97)	4	3224.4
plus $eAER_{Ellam}$	2.67 (2.42,2.93)	4	3224.7
plus $eAER_{Ix}$	2.73 (2.48,3.00)	4	3240.2
plus $eAER_{Walser}$	2.72 (2.47,2.99)	4	3242.6
plus ACR and $eAER_{Ellam}$		5	3224.7
plus ACR and $eAER_{Ix}$		5	3245.0
plus ACR and $eAER_{Walser}$		5	3244.9

ACR=albumin:creatinine ratio. eAER=estimated albumin excretion rate. eGFR=estimated glomerular filtration rate. §Compared to null model. These analyses are based on 5522 participants as 30 of the 5552 participants with complete information on ACR and eGFR had missing values of weight at baseline, and therefore the values of $eAER_{Ix}$ and $eAER_{Walser}$ could not be calculated for these individuals.

Table S2: Relative ability of each marker of albuminuria to predict progression to ESRD as a percentage of the informativeness of ACR and eAER combined, by age, sex, ethnicity, weight, cause of kidney disease and eGFR

		Relative informativeness* for prediction of ESRD		
	**n**	**Both ACR and eAER**	**ACR only**	**eAER only**
Age (years)				
40 to 49	985	100% (χ^2=62)	98% (χ^2=61)	98% (χ^2=61)
50 to 59	1336	100% (χ^2=64)	96% (χ^2=61)	96% (χ^2=61)
60 to 69	1547	100% (χ^2=175)	100% (χ^2=175)	100% (χ^2=175)
≥70	985	100% (χ^2=62)	98% (χ^2=61)	98% (χ^2=61)
Sex				
Male	3464	100% (χ^2=345)	100% (χ^2=345)	100% (χ^2=345)
Female	2088	100% (χ^2=112)	100% (χ^2=112)	100% (χ^2=112)
Ethnicity				
White	3925	100% (χ^2=246)	100% (χ^2=246)	100% (χ^2=246)
Asian	1426	100% (χ^2=231)	100% (χ^2=231)	100% (χ^2=230)
Weight (kg)				
<70	1831	100% (χ^2=193)	100% (χ^2=193)	100% (χ^2=192)
≥70 to <85	1867	100% (χ^2=139)	99% (χ^2=137)	99% (χ^2=138)
≥85	1824	100% (χ^2=120)	99% (χ^2=119)	99% (χ^2=118)
eGFR (mL/min/1.73m²)				
≥30	1775	100% (χ^2=125)	98% (χ^2=123)	98% (χ^2=123)
<30	3777	100% (χ^2=330)	100% (χ^2=329)	100% (χ^2=330)
Diastolic blood pressure (mm Hg)				
<80	2607	100% (χ^2=213)	98% (χ^2=210)	99% (χ^2=211)
≥80 to <90	1708	100% (χ^2=102)	99% (χ^2=100)	98% (χ^2=100)
≥90 to <100	899	100% (χ^2=78)	98% (χ^2=76)	99% (χ^2=77)
≥100	323	100% (χ^2=28)	100% (χ^2=27)	100% (χ^2=28)
Systolic blood pressure (mm Hg)				
<140	2887	100% (χ^2=128)	100% (χ^2=128)	100% (χ^2=127)
≥140 to <160	1723	100% (χ^2=168)	100% (χ^2=167)	100% (χ^2=168)
≥160 to <180	715	100% (χ^2=103)	100% (χ^2=103)	100% (χ^2=103)
≥180	220	100% (χ^2=24)	97% (χ^2=23)	98% (χ^2=23)
Cause of kidney disease				
Glomerulonephritis	973	100% (χ^2=118)	98% (χ^2=117)	98% (χ^2=116)
Diabetic nephropathy	792	100% (χ^2=152)	99% (χ^2=151)	100% (χ^2=152)
Cystic kidney disease	614	100% (χ^2=4.6)	67% (χ^2=3.1)	70% (χ^2=3.2)
Other diagnoses	2959	100% (χ^2=315)	100% (χ^2=314)	100% (χ^2=315)
All participants	5552	100% (χ^2=441)	100% (χ^2=441)	100% (χ^2=441)

ACR=albumin:creatinine ratio. eAER=estimated albumin excretion rate. *Informativeness of the indicator of albuminuria (as indicated by the χ^2 statistic giving the improvement in fit compared to a model containing age, sex and estimated glomerular filtration rate), as a percentage of the informativeness of ACR and eAER combined.

Table S3: Comparison of predictive strengths for ESRD relative risk of different indicators of albuminuria, using Fine and Gray regression.

	Relative risk per 10 fold increase in indicator of albuminuria (95% CI)	Degrees of freedom (df)	Improvement in fit (χ^2)§
Null model		0	0
plus ACR	3.18 (2.92,3.46)	1	822.5
plus $eAER_{Ellam}$	3.04 (2.80,3.30)	1	806.5
plus $eAER_{Ix}$	3.08 (2.83,3.35)	1	803.0
plus $eAER_{Walser}$	3.10 (2.85,3.37)	1	823.0
plus ACR and $eAER_{Ellam}$		2	822.5
plus ACR and $eAER_{Ix}$		2	822.5
plus ACR and $eAER_{Walser}$		2	828.7
Age and sex		2	200.4
plus ACR	3.00 (2.75,3.28)	3	917.7
plus $eAER_{Ellam}$	2.96 (2.72,3.23)	3	917.1
plus $eAER_{Ix}$	3.01 (2.76,3.28)	3	921.2
plus $eAER_{Walser}$	2.98 (2.74,3.25)	3	918.1
plus ACR and $eAER_{Ellam}$		4	917.8
plus ACR and $eAER_{Ix}$		4	921.7
plus ACR and $eAER_{Walser}$		4	920.9
Age, sex and eGFR		3	2474.8
plus ACR	1.99 (1.78,2.22)	4	2708.2
plus $eAER_{Ellam}$	1.97 (1.77,2.20)	4	2709.2
plus $eAER_{Ix}$	2.05 (1.83,2.29)	4	2728.5
plus $eAER_{Walser}$	2.05 (1.84,2.29)	4	2733.7
plus ACR and $eAER_{Ellam}$		5	2711.2
plus ACR and $eAER_{Ix}$		5	2755.3
plus ACR and $eAER_{Walser}$		5	2756.2

ACR=albumin:creatinine ratio. eAER=estimated albumin excretion rate. eGFR=estimated glomerular filtration rate. §Compared to null model. These analyses are based on 5522 participants as 30 of the 5552 participants with complete information on ACR and eGFR had missing values of weight at baseline, and therefore the values of $eAER_{Ix}$ and $eAER_{Walser}$ could not be calculated for these individuals.

Table S4: Comparison of predictive strengths for vascular event relative risk of different indicators of albuminuria, using alternative equations to estimate creatinine excretion rate.

	Relative risk per 10 fold increase in indicator of albuminuria (95% CI)	Degrees of freedom (df)	Improvement in fit (χ^2)§
Established risk factors*		32	674.6
plus ACR	1.53 (1.38,1.69)	33	741.6
plus $eAER_{Ellam}$	1.52 (1.37,1.68)	33	741.8
plus $eAER_{Ix}$	1.52 (1.37,1.68)	33	740.0
plus $eAER_{Walser}$	1.51 (1.36,1.67)	33	739.7
plus ACR and $eAER_{Ellam}$		34	742.0
plus ACR and $eAER_{Ix}$		34	742.2
plus ACR and $eAER_{Walser}$		34	742.0
Established risk factors* and eGFR		33	744.0
plus ACR	1.36 (1.22,1.52)	34	774.1
plus $eAER_{Ellam}$	1.36 (1.22,1.52)	34	774.4
plus $eAER_{Ix}$	1.35 (1.21,1.51)	34	773.6
plus $eAER_{Walser}$	1.35 (1.21,1.51)	34	773.6
plus ACR and $eAER_{Ellam}$		35	774.9
plus ACR and $eAER_{Ix}$		35	774.3
plus ACR and $eAER_{Walser}$		35	774.2

ACR=albumin:creatinine ratio. eAER=estimated albumin excretion rate. eGFR=estimated glomerular filtration rate. §Compared to null model. *The established vascular risk factors included are: age, sex, ethnicity, country, systolic blood pressure, diastolic blood pressure, LDL cholesterol, HDL cholesterol, smoking status, prior diabetes and prior vascular disease. These analyses are based on 5509 participants as 26 of the 5535 participants included in the analyses of VE risk had missing values of weight at baseline, and therefore the values of $eAER_{Ix}$ and $eAER_{Walser}$ could not be calculated for these individuals.

Table S5: Comparison of predictive strengths for mortality relative risk of different indicators of albuminuria, using alternative equations to estimate creatinine excretion rate.

	Relative risk per 10 fold increase in indicator of albuminuria (95% CI)	Degrees of freedom (df)	Improvement in fit (χ^2)§
Vascular mortality			
Established risk factors* and eGFR		33	427.9
plus ACR	1.63 (1.32,2.01)	34	449.1
plus $eAER_{Ellam}$	1.61 (1.31,1.98)	34	448.8
plus $eAER_{Ix}$	1.61 (1.31,1.99)	34	448.8
plus $eAER_{Walser}$	1.59 (1.30,1.96)	34	447.9
plus ACR and $eAER_{Ellam}$		35	451.1
plus ACR and $eAER_{Ix}$		35	449.2
plus ACR and $eAER_{Walser}$		35	450.0
Non-vascular mortality			
Established risk factors* and eGFR		33	560.0
plus ACR	1.51 (1.29,1.77)	34	586.4
plus $eAER_{Ellam}$	1.49 (1.28,1.75)	34	585.5
plus $eAER_{Ix}$	1.47 (1.26,1.72)	34	583.7
plus $eAER_{Walser}$	1.45 (1.24,1.70)	34	582.4
plus ACR and $eAER_{Ellam}$		35	594.6
plus ACR and $eAER_{Ix}$		35	593.2
plus ACR and $eAER_{Walser}$		35	597.3
All-cause mortality			
Established risk factors* and eGFR		33	1037.7
plus ACR	1.55 (1.37,1.74)	34	1090.5
plus $eAER_{Ellam}$	1.53 (1.36,1.72)	34	1089.3
plus $eAER_{Ix}$	1.51 (1.34,1.70)	34	1086.1
plus $eAER_{Walser}$	1.49 (1.33,1.68)	34	1083.6
plus ACR and $eAER_{Ellam}$		35	1100.8
plus ACR and $eAER_{Ix}$		35	1100.6
plus ACR and $eAER_{Walser}$		35	1107.1

ACR=albumin:creatinine ratio. eAER=estimated albumin excretion rate. eGFR=estimated glomerular filtration rate. §Compared to null model. *The established risk factors for mortality included are: age, sex, ethnicity, country, systolic blood pressure, diastolic blood presure, LDL cholesterol, HDL cholesterol, smoking status, prior diabetes and prior vascular disease. These analyses are based on 5509 participants as 26 of the 5535 participants included in the analyses of mortality risk had missing values of weight at baseline, and therefore the values of $eAER_{Ix}$ and $eAER_{Walser}$ could not be calculated for these individuals.
