# Supplementary material for: Prognostic utility of estimated albumin excretion rate in chronic kidney disease: results from the Study of Heart and Renal Protection
Source: Nephrol Dial Transplant. 2017 Jan 14;33(2):257–64. doi: 10.1093/ndt/gfw396 (PMC5837220; doi:10.1093/ndt/gfw396)
Supplement: Supplementary Technical appendix [file gfw436_technical_appendix_clean_280816.docx]

**Technical appendix**

*Appendix A1: Systematic review of the literature.*

Our study design included discrete choice experiments, conjoint analyses, adaptive conjoint analysis, and best-worst scaling. The eligibility criteria for inclusion in the review is that the CA, DCE, or ACA study must have been published and conducted since 1990, and the analysis must present findings from the empiric analysis of DCE, CA, or ACA data (i.e. excluding study protocols, reviews, editorials or opinion pieces).

The information sources and databases used to obtain citations for potentially relevant studiesincluded Pubmed (Medline), and Embase, Psychinfo, and Econlit, with additional handsearching of reference lists of included studies, and direct contact with study authors. One study not identified in the electronic databases[1] was subsequently included. The last set of searches were conducted in December 2015. The search strategy used for the Pubmed search included the following search terms:

#1: Conjoint Analysis

#2: Discrete Choice

#3: Kidney OR Nephrology OR Renal

#4: #1 OR #2

#5: #3 AND #4

The approach to study selection involved review of abstracts and obtaining any potentially relevant studies relating to the study inclusion criteria. This required that the research needed to relate primarily to a Nephrology application and that the analysis must contain econometric analysis of DCE, CA, or ACA data. It was not necessary to include search terms relating to ‘Best Worst Scaling (BWS)’ because in common with earlier systematic reviews of the DCE, CA, or ACA literature,[2, 3] we only wanted to include BWS studies if they also used DCE, CA, or ACA methodologies. This means that relevant BWS studies would be picked up by DCE, CA, or ACA related search terms.

Other analyses (for example that purely outlined DCE, CA, or ACA protocols) might be cited but would not be systematically reviewed. The approach used to collect data was to establish how each of the studies met the criteria which are set out in tables 1 to 15 within the electronic supplements. The data items) which included variables and review criteria for which data were collected are set out in tables 1 to 15. When appropriate we explain and define these variables / criteria within tables or the explanatory test within this appendix.

*Appendix A2: Evaluation of the choice study methodology*

As a starting point for establishing criteria against which to systematically review these renal / nephrology DCE, CA, or ACA studies we combined the review criteria used in an earlier systematic review of the healthcare DCE, and CA literature,[3] and those used in an systematic review of the DCE, CA, and ACA literature.[2] Although the latter incorporated review criteria deployed in the earlier review,[3] it also established additional information about some methods used relating to preference heterogeneity.^^[[1]](#footnote-1)^^ The specification of some of these review criteria[2] was informed by other highly regarded review criteria[4] for DCE, and CA literature. In the present review we expanded the Clark et al (2014)[2] criteria to encompass a range of other relevant systematic review criteria. Tables 1 – 15 summarising how each of the 143 studies selected performed against the full range of review criteria deployed are provided within these electronic supplements.

Table 1 provides information about whether DCEs used non-labelled choice or labelled choice designs. Overall 11 used non-labelled choice[1, 5-7] DCE designs, with only 3 DCEs using labelled choice designs.[8-10] Information is also collated about the number of attributes used in a DCE. Overall 2 studies[9, 10] had between 2 and 3 attributes; 1 study had between 4 and 5 attributes;[8] 4 studies had 6 attributes;[1, 5-7, 11-14] 4 studies had between 7 and 9 attributes;[11-14] 2 studies had 10 attributes;[15, 16] and 1 study had more than 10 attributes.[17]

The DCE attributes covered a range of domains (table 3 2). Overall 4 involved a monetary measure; 13 involved a domain relating to time; 8 involved a domain relating to risk; 12 had a domain relating to health status; 14 involved a domain relating to health care; and 12 involved a domain relating to ‘Other’. It should be noted that sometimes an attribute related to more than 1 domain. For example one analysis contained an attribute described as ‘Risk of HIV infection’,[10] which related to both risk and health status. Table 3 2 provided some information about how the surveys were administered. In some cases DCE surveys used more than 1 form of administration. Overall, 7 involved self-completed questionnaires; 5 involved interview administered questionnaires; 6 involved computerized reviews or computer reviews; and 1 did not clearly report how the survey was administered.

Table 3 provides information relating to the number of alternatives in each DCE. Overall 10 studies involved a choice of 1 out of 2 options, and 4 analyses involved a choice of 1 out of 3 options. In total 13 analyses involved Discrete Choice Experiment or Conjoint Analysis methods. Only one analysis[16] involved an adaptive conjoint analysis, and none of the analyses involved ‘Best-Worst-Scaling’ methods. Overall no analyses involved respondents making fewer than 8 choices; 10 analyses involved respondents making between 9 and 16 choices; 3 analyses involved making over 13 choices; whilst in 1 case[16] it was unclear how many choices respondents faced.

Table 4 provides further information relating to the choice survey design. Just 1 of the analyses involved a full factorial design;[9] 12 involved a fractional factorial design; and in 1 analysis the design was not clearly reported.[10] Overall 8 analyses used a main effects design; 4 analyses indicated they used a design catering for both main effects and 2 way interactions; in one case[9] a design plan was not applicable as the full factorial was used; and in 1 case the design plan was not clearly reported.[10] Overall 8 analyses used a software package; 4 analyses said they used an ‘other’ design source; 1 analysis[10] provided no further details; and in 1 case a design was not required because the full factorial was used.[9]

Table 5 provides further information about the source of designs. 5 analyses reported they used Ngene software;[18] 7 analyses reported they used ‘Other.’ Of these, 3 used Street and Burgess designs,[1, 5, 6] one of them used the full factorial,[9] one of them used Sloans library of orthogonal arrays,[7] one of them used Gauss,[12] and the other used an ACBC (Adaptive Conjoint Based Analysis design);[16] 2 analyses did not indicate the design source, and no studies used SPEED, SPSS, SAS, or Sawtooth software.

Table 6 provides further details of the design source, and methods used to create choice sets, if reported. 1 analysis used a website; 3 used an expert design source (Street and Burgess),[19] in 2 cases the design source was not clearly reported, and no analyses used a ‘Catalogue’. 3 reported use of orthogonal arrays with foldover, no analyses reported use of orthogonal arrays with single profiles; orthogonal arrays with random pairing; orthogonal arrays paired with a constant comparator; or orthogonal arrays foldover with random pairing.

Table 7 provides further information about the methods used to create choice sets. 5 used D-efficient orthogonal arrays generated using Ngene software;[18] 1 used a D-efficient design generated using Gauss,[12] 1 used orthogonal arrays which are pragmatically chosen; 1 used other forms of D-efficient orthogonal arrays; and 2 used ‘Other’ methods to create choice sets including the full factorial[9] and Adaptive Conjoint Analysis.[16]

Table 8 provides information about the econometric estimation procedures used to analyse data. It should be noted that some analyses used more than one estimation method. Overall 2 analyses used Random Effects Probit; 2 analyses used Logit; 2 analyses used conditional logit / multinomial logit; 7 used Mixed Logit / Random Parameters Logit models. No analyses used Probit, Random Effects Logit, or Nested logit models.

Table 9 provides further information about the econometric estimation procedures used. Overall 1 analysis used Latent Class Models[11] and this was alongside Mixed Logit; and 1 used an ‘Other’ estimation method[16] which involved the use of the software package ACBC to calculate part-worth utilities. No analyses used Generalized Mixed Logit (G-MXL); or Generalized Multinomial Logit (G-MNL). Table 11 also provides information about whether (if preference heterogeneity models such as Mixed Logit, Random Parameter Logit, or Latent Class Models were applied) there was evidence of preference heterogeneity. Our review found in all 7 analyses (involving either Mixed Logit, Random Parameter Logit, or Latent Class Models, or more than 1 of these) there was evidence of statistically significant preference heterogeneity. Only 4 / 7 of the studies involving Mixed Logit or Random Parameter Logit provided information about the number of replications (sometimes known as Halton draws)[20] assumed for these analyses. Also 5 / 7 analyses reported they had used normal distributions relating to the distribution of preference heterogeneity, although one of these reported that a log distribution had been assumed at the same time (e.g. in relation to the distribution of price).[9] No analyses suggested any other distributional assumptions had been made for Mixed Logit models.

Table 10 summarizes information about whether methods to explore preference heterogeneity had been used and in all cases such methods had been deployed. The table then describes the methods used for each of these analyses.

Table 11 relates to whether ‘Validity tests’ had been deployed and if so which ones. Findings indicated that 14 analyses involved tests of internal theoretical validity (e.g. they assessed whether some results seemed to be in line with prior expectations about the signs of coefficients etc); and 1 analysis involved an internal test of ‘non-satiation’, and one analysis involved a test of compensatory decision making. No analyses involved a test of external validity, or an internal test of transitivity, or an internal test of ‘Sens expansion and contraction criteria’. In terms of qualitative validity tests 9 analyses used qualitative methods to inform attribute selection, and 9 used qualitative methods to inform level selection. 11 analyses used pre-testing pilot questionnaires.

Table 12 provides further information relating to validity tests. Overall 6 analyses reported that they used ‘Other’ validity tests. This included one analysis[8] which claimed to check respondent adherence to monotonicity; another analysis[9] which collated information about respondents’ self-perceived difficulty in completing DCE questionnaires; one analysis which used a ‘think-aloud’ exercise during piloting of the questionnaire to identify whether respondents deployed simplifying heuristics;[12] two which used a practice question[13, 14] before the main 12 DCE questions, and secondary analysis for respondents answering the practice question ‘correctly’[14]; also one analysis[7] considered DCE results alongside a ranking of adverse events (in order of severity) which had been undertaken by doctors and nurses. Overall 3 analyses[12-14] reported that they used qualitative methods such as de-briefing choices to strengthen respondents’ understanding. Reassuringly no analyses failed to conduct any form of validity test.

Table 13 collates information about the main objectives and output of DCEs. In contrast to table 1 2 in the main text of the paper that presents similar information, an attempt is made in table 14 13 to identify the main objective and output of the DCE (as opposed to a range of objectives and outputs, as with table 1). Findings suggest that 5 analyses had a main objective relating to category C (Investigating trade-offs between health outcomes and patient or consumer experience factors); 6 analyses had a main objective relating to category F (Developing priority setting frameworks); 3 analyses had a main objective relating to category G (Health professionals preference for treatment or screening options for patients); finally 1 analysis had a main objective relating to category H (Other). One study had two main objectives[7] relating to both category C (Investigating trade-offs between health outcomes and patient or consumer experience factors) and category G (Health professionals preferences for treatment or screening options for patients). No analyses had a main objective relating to category A (Patient consumer experience), or category B (Valuing Health Outcomes), or category D (Estimating utility weights within the QALY framework), or category E (Job choices).

Table 14 collates information about how the key findings were presented. Overall 1 analysis presented results in terms of ‘Per willingness to pay unit’; 2 analyses presented results in terms of ‘Per time period’; 8 analyses presented results in terms of an ‘Odds ratio’; and 9 analyses used an ‘Other’ outcome measure. This included an analysis which presented results in terms of ‘rank order importance of attributes’[6]; an analysis which presented results in terms of willingness to switch dialysis[8]; an analysis which considered whether a new policy was preferred to the current one[11] an analysis which looked at the odds ratio of receiving an organ;[17] an analysis which used marginal rate of substitution with respect to progression-free survival;[12] an analysis which looked at willingness to trade life expectancy to improve another attribute[13]; an analysis which established whether conservative care of dialysis was preferred, using an odds ratio[14]; an analysis which considered the relative importance of attributes and marginal rates of substitution between attributes[7]; and an analysis which considered willingness to accept a kidney from a donor at increased risk of blood borne viral infection [DIRVI][10]. No analyses presented results in terms of a ‘Monetary welfare measure’, or a ‘Utility score’, or a ‘Per risk unit’, or a ‘probability score’.

Finally in table 15 we extracted information about how studies might have catered for population diversity with respect to ethnicity or language.

*Appendix A.3. Discrete choice experiment models for analysis.*

*Random Utility Theory (RUT):* DCEs are usually grounded in random utility theory [RUT].[21] This assumes that demand can be decomposed into a systematic component for estimation, and a random component which can controlled for (e.g. using Random Effects models).

Sometimes the terms DCE and CA are used inter-changeably. Moreover, many analyses described as CA could be described as DCEs, if they have foundations in both the theory of demand[22] and RUT.[21] However a proportion of analyses described as CA may deploy econometric methods incompatible with random utility theory,[21] so they can be described as CA, but not DCEs. Adaptive Conjoint Analyses (ACA) are also grounded in the theory of demand.[22] However, they adopt an adaptive procedure for framing questions whereby the sequence of questions is based upon earlier responses (generated by a computer programme). Moreover, the data generated is subject to analysis whereby ‘part-worth’ utilities are calculated.[16]

*Preference heterogeneity models:* It is likely that individuals will have different preferences, and that some of the preference heterogeneity is unrelated to observable personal characteristics. This issue cannot be investigated using the traditional modelling tools. Mixed Logit (sometime called Random Parameter Logit) and latent class logit models can instead be used to analyse the data from the choice experiment. Mixed and latent class logit models are extensions of the standard logit model that allow us to estimate the distribution of preferences for the attributes in the experiment.[23]

*Mixed Logit Models (sometimes known as random parameter logit [RPL] models):* These allow consideration of the full distribution of a parameter estimate, and the fixed parameter becomes a random parameter. ‘Random parameter’ simply implies that each individual has an associated parameter estimate on that specified distribution,[24] so a parametric distribution is assumed. In this way a point estimate of a parameter can be generated together with information about the standard deviation from the point estimate (based upon an assumed distribution for the random parameter). Particular care however needs to be applied when selecting a suitable distribution for the random parameter (normally either a normal distribution or log distribution is assumed), because otherwise the results generated may simply be an artefact of inappropriate distributional assumptions that have been applied.

*Latent Class Models (LCM):*  The underlying theory of latent class models suggests that individuals’ choice behaviour and preferences can be allocated into a set of *Q* latent classes. Preferences within each class are assumed to be homogenous, but allowed to differ across classes.[25] In contrast to Mixed Logit preference heterogeneity models, latent class models are non-parametric.

**References.**

1. Clark MD, Gumber AK, Leech D*, et al.* Prioritising patients for renal transplantation? Analysis of patient preferences for kidney allocation according to ethnicity and gender. Diversity in Health & Care 2009;6(3)

2. Clark MD, Determann D, Petrou S*, et al.* Discrete choice experiments in health economics: a review of the literature. Pharmacoeconomics 2014;32(9):883-902

3. de Bekker-Grob EW, Ryan M, Gerard K. Discrete choice experiments in health economics: a review of the literature. Health Econ 2012;21(2):145-172

4. Bridges JF, Hauber AB, Marshall D*, et al.* Conjoint analysis applications in health--a checklist: a report of the ISPOR Good Research Practices for Conjoint Analysis Task Force. Value Health 2011;14(4):403-413

5. Clark MD, Leech D, Gumber A*, et al.* Who should be prioritized for renal transplantation?: Analysis of key stakeholder preferences using discrete choice experiments. BMC Nephrol 2012;13:152

6. Davison SN, Kromm SK, Currie GR. Patient and health professional preferences for organ allocation and procurement, end-of-life care and organization of care for patients with chronic kidney disease using a discrete choice experiment. Nephrol Dial Transplant 2010;25(7):2334-2341

7. Park MH, Jo C, Bae EY*, et al.* A comparison of preferences of targeted therapy for metastatic renal cell carcinoma between the patient group and health care professional group in South Korea. Value Health 2012;15(6):933-939

8. Halpern SD, Berns JS, Israni AK. Willingness of patients to switch from conventional to daily hemodialysis: looking before we leap. Am J Med 2004;116(9):606-612

9. Kjaer T, Bech M, Kronborg C*, et al.* Public preferences for establishing nephrology facilities in Greenland: estimating willingness-to-pay using a discrete choice experiment. Eur J Health Econ 2013;14(5):739-748

10. Reese PP, Tehrani T, Lim MA*, et al.* Determinants of the decision to accept a kidney from a donor at increased risk for blood-borne viral infection. Clin J Am Soc Nephrol 2010;5(5):917-923

11. Howard K, Jan S, Rose JM*, et al.* Preferences for Policy Options for Deceased Organ Donation for Transplantation: A Discrete Choice Experiment. Transplantation 2015

12. Mohamed AF, Hauber AB, Neary MP. Patient benefit-risk preferences for targeted agents in the treatment of renal cell carcinoma. Pharmacoeconomics 2011;29(11):977-988

13. Morton RL, Snelling P, Webster AC*, et al.* Dialysis modality preference of patients with CKD and family caregivers: a discrete-choice study. Am J Kidney Dis 2012;60(1):102-111

14. Morton RL, Snelling P, Webster AC*, et al.* Factors influencing patient choice of dialysis versus conservative care to treat end-stage kidney disease. Clin J Am Soc Nephrol 2012;184(5):E277-283

15. Foote C, Morton RL, Jardine M*, et al.* COnsiderations of Nephrologists when SuggestIng Dialysis in Elderly patients with Renal failure (CONSIDER): a discrete choice experiment. Nephrol Dial Transplant 2014;29(12):2302-2309

16. Whitman CB, Shreay S, Gitlin M*, et al.* Clinical factors and the decision to transfuse chronic dialysis patients. Clin J Am Soc Nephrol 2013;8(11):1942-1951

17. Howard K, Jan S, Rose JM*, et al.* Community preferences for the allocation of donor organs for transplantation: a discrete choice study. Transplantation 2015;99(3):560-567

18. Rose JM BM. Ngene. Available from: <http://www.choice-metrics>. com/download.html 2016

19. Street DJ BL, Louviere J,. Quick and easy choice sets: constructing optimal and nearly optimal stated choice experiments. Int J Res Mark 2005;22:459-470

20. Hensher DA RJ, Greene WH,. Applied Choice Analysis. A Primer. . Cambridge University Press. New York 2005:616

21. McFadden D. Computing Willingness to Pay in Random Utility Models. Trade theory and econometrics. Essays in honour of John S Chipman. Studies in the Modern World Economy 1999;Chapter 15

22. Lancaster KJ. New Approach to Consumer Theory. Journal of Political Economy 1966;74(2):132-157

23. Hole A. Modelling heterogeneity in patients' preferences for the attributes of a general practitioner appointment. CHE Research paper 22, University of York 2007;January

24. Howard K, Jan S, Rose J*, et al.* Community Preferences for the Allocation & Donation of Organs--the PAraDOx Study. BMC Public Health 2011;11:386

25. Erdem S, Thompson C. Prioritising health service innovation investments using public preferences: a discrete choice experiment. BMC Health Serv Res 2014;14:360

1. Information was compiled for any mixed logit or random parameter logit analyses conducted (e.g. whether details of the number of Halton draws / replications were provided, plus details of whether distributional assumptions made in relation to the distribution of preference heterogeneity were made explicit). [↑](#footnote-ref-1)
